# *Leishmania infantum* Lipophosphoglycan-Deficient Mutants: A Tool to Study Host Cell-Parasite Interplay

**DOI:** 10.3389/fmicb.2018.00626

**Published:** 2018-04-05

**Authors:** Milena Lázaro-Souza, Christine Matte, Jonilson B. Lima, Guillermo Arango Duque, Graziele Quintela-Carvalho, Áislan de Carvalho Vivarini, Sara Moura-Pontes, Cláudio P. Figueira, Flávio H. Jesus-Santos, Ulisses Gazos Lopes, Leonardo P. Farias, Théo Araújo-Santos, Albert Descoteaux, Valéria M. Borges

**Affiliations:** ^1^Laboratory of Inflammation and Biomarkers, Gonçalo Moniz Institut, Oswaldo Cruz Foundation, Salvador, Brazil; ^2^Department of Legal Medicine, Federal University of Bahia, Salvador, Brazil; ^3^Institut National de la Recherche Scientifique –Institut Armand-Frappier, Laval, QC, Canada; ^4^Center of Biological Sciences and Health, Federal University of Western of Bahia, Barreiras, Brazil; ^5^Instituto Federal de Educação, Ciência e Tecnologia Baiano (IFBaiano), Alagoinhas, Brazil; ^6^Laboratory of Molecular Parasitology, Carlos Chagas Filho Biophysics Institute, Center of Health Science, Universidade Federal do Rio de Janeiro, Rio de Janeiro, Brazil

**Keywords:** Lipophosphoglycan, *Leishmania infantum*, gene targeting, lipid droplets, macrophage

## Abstract

Lipophosphoglycan (LPG) is the major surface glycoconjugate of metacyclic *Leishmania* promastigotes and is associated with virulence in various species of this parasite. Here, we generated a LPG-deficient mutant of *Leishmania infantum*, the foremost etiologic agent of visceral leishmaniasis in Brazil. The *L. infantum* LPG-deficient mutant (Δ*lpg1*) was obtained by homologous recombination and complemented via episomal expression of *LPG1* (Δ*lpg1* + *LPG1*). Deletion of *LPG1* had no observable effect on parasite morphology or on the presence of subcellular organelles, such as lipid droplets. While both wild-type and add-back parasites reached late phase in axenic cultures, the growth of Δ*lpg1* parasites was delayed. Additionally, the deletion of *LPG1* impaired the outcome of infection in murine bone marrow-derived macrophages. Although no significant differences were observed in parasite load after 4 h of infection, survival of Δ*lpg1* parasites was significantly reduced at 72 h post-infection. Interestingly, *L*. *infantum* LPG-deficient mutants induced a strong NF-κB-dependent activation of the inducible nitric oxide synthase (iNOS) promoter compared to wild type and Δ*lpg1* + *LPG1* parasites. In conclusion, the *L. infantum* Δ*lpg1* mutant constitutes a powerful tool to investigate the role(s) played by LPG in host cell-parasite interactions.

## Introduction

Lipophosphoglycan (LPG) is one of the most abundant components of *Leishmania* membranes (Turco and Descoteaux, 1992). In the course of parasite interaction with invertebrate hosts, LPG binds to the midgut epithelium of specific species of the sandfly vectors (Sacks et al., 2000), and protects parasites against the digestive enzymes present in the peritrophic matrix following blood feeding (Sacks and Kamhawi, 2001). In vertebrate hosts, LPG contributes to virulence by shielding *Leishmania* against the complement system (Spath et al., 2003) and by inhibiting phagolysosomal biogenesis (Desjardins and Descoteaux, 1997; Vinet et al., 2009; Moradin and Descoteaux, 2012). Purified LPG has been considered as a pathogen-associated molecular pattern molecule (PAMP) that triggers Toll-like receptors (TLR) and is also known to interfere with pro-inflammatory and signaling pathways in host cells (Descoteaux et al., 1991; Descoteaux and Turco, 1993; Becker et al., 2003; de Veer et al., 2003; Kavoosi et al., 2009; Rojas-Bernabé et al., 2014; Tavares et al., 2014; Lima et al., 2017).

This complex glycolipid is organized in four domains: a conserved 1-O-alkyl-2-lyso-phosphatidyl(myo)inositol membrane anchor, a conserved diphosphoheptasaccharide core structure, a polymer of repeating phosphodisaccharide units (phosphoglycan or PG) carrying species-specific side chains and variable, often mannose-rich cap structures (Turco and Descoteaux, 1992; McConville and Ferguson, 1993). Although the biosynthesis of LPG has attracted considerable interest, to date only few enzymes and transporters involved in this process have been identified either biochemically, genetically, or both (Ryan et al., 1993; Descoteaux et al., 1995, 1998, 2002).

One of the key enzymes in the biosynthesis of LPG is *LPG1*, a putative galactofuranosyl transferase specifically involved in the synthesis of the LPG glycan core (Ryan et al., 1993). Consequently, parasites lacking the *LPG1* gene (Δ*lpg1*) express a truncated LPG without the PG domain; they nonetheless assemble and secrete other PG-containing molecules (Dermine et al., 2000; Späth et al., 2000). Both *L. major* and *L. donovani* require *LPG1* for the establishment of infection within macrophages, as evidenced by the elimination of *LPG1*-null mutants following phagocytosis; yet, restoration of LPG expression by genetic complementation restored the capacity to replicate within macrophages (Späth et al., 2000; Lodge et al., 2006). Interestingly, phosphoglycan synthesis does not seem to be an absolute requirement for virulence in all *Leishmania* species, since *L. mexicana* phosphoglycan-deficient parasites were found to be similarly virulent to their wild-type (WT) counterparts (Ilg et al., 1999, 2001; Ilg, 2000). This difference in LPG requirement for the establishment of infection within macrophages may be related to the fact that *L. mexicana* resides in large fusogenic communal vacuoles, as opposed to the non-fusogenic, tight individual vacuoles in which *L. major* and *L. donovani* replicate. The role played by *LPG1* in *L. infantum* infectivity in mammals remains to be established.

This report describes the disruption of *LPG1* in *L. infantum*, the main etiological agent of visceral leishmaniasis in Brazil. While deletion of *LPG1* did not alter parasite morphology *in vitro* or the presence of subcellular organelles, e.g., lipid droplets (LD), Δ*lpg1* parasites experienced distinct infection outcomes in comparison to WT parasites. Hence, the *L. infantum LPG1*-null strain described in the present study constitutes a powerful tool to investigate the role of LPG in host-parasite interactions.

## Methods

### Ethics statement

This study was carried out in accordance with the recommendations of Institutional Review Board for Animal Experimentation (CEUA), Gonçalo Moniz Institute, Fundação Oswaldo Cruz. The protocol was approved by the Institutional Review Board for Animal Experimentation (CEUA), Gonçalo Moniz Institute, Fundação Oswaldo Cruz (Protocol No. 021/2015).

### Animals

Inbred male C57BL/6 mice, aged 6–8 weeks, were obtained from the animal care facility of the Gonçalo Moniz Institute, Fundação Oswaldo Cruz (IGM-FIOCRUZ, Bahia, Brazil).

### Targeted deletion of the *LPG1* gene and complementation

**Figure 1:**
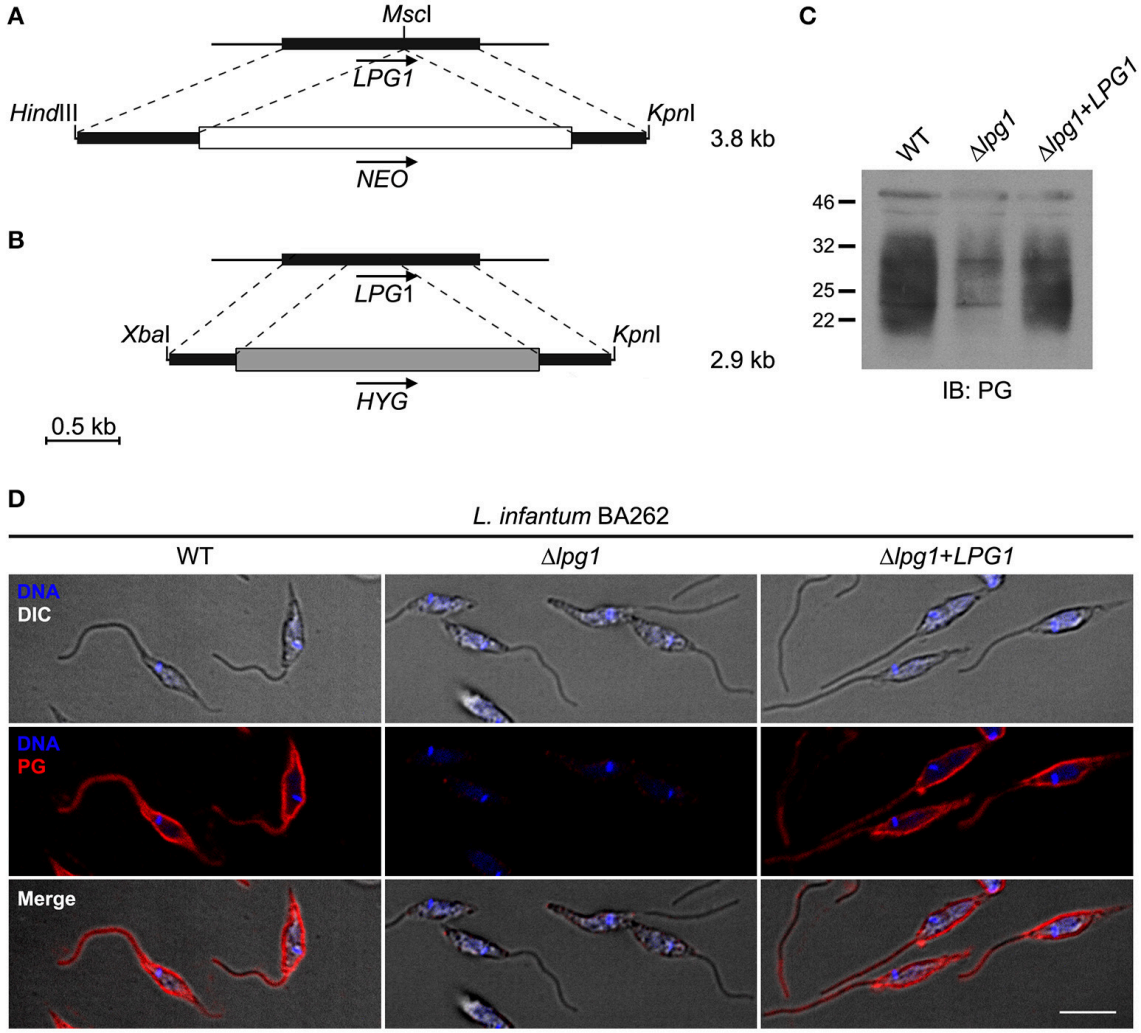
Constructs for the targeted deletion and complementation of the *LPG1* gene in *Leishmania infantum*. **(A,B)** *LPG1*::*NEO* and *LPG1*::*HYG* targeting constructs for the disruption of *LPG1*. For the *LPG1*::*NEO* construct, the *NEO* resistance cassette (white box) was inserted in the *Msc*I site of the *LPG1* ORF (black rectangle). In the *LPG1*::*HYG* construct, portions of the *LPG1* ORF (black rectangles) corresponding to positions +1 to +437 and to positions +781 to +1247 downstream of the ATG translation initiation codon flank the HYG resistance cassette (shaded rectangle). Dashed lines delimit regions of recombination between the LPG1 gene and the targeting constructs. Arrows indicate gene orientation. **(C)** Western blot analysis of LPG expression in WT, Δ*lpg1*, and Δ*lpg1* + *LPG1* promastigotes. Parasite lysates were probed with the anti-phosphoglycan (PG) antibody CA7AE, as described in Materials and Methods. **(D)** Confocal immunofluorescence analysis of WT, Δ*lpg1* and Δ*lpg1* + *LPG1* parasites. Late log-phase promastigotes were adhered on Poly-L-Lysine-coated glass coverslips, fixed and incubated with DAPI to stain DNA (blue), and with the CA7AE antibody to visualize LPG and other Gal(β1,4)Man(α1-PO4) repeating unit-containing PGs (red), as described in Materials and Methods. Fluorescence staining images merged with differential interference contrast (DIC) are shown in the lower panels. Scale bar, 5 μm.

The constructs for *LPG1* (beta galactofuranosyl transferase) gene targeting were designed based on the *L. infantum LPG1* gene sequence (GenBank accession No. GU233511). Homozygous *LPG1*-null mutants (Δ*lpg1*) were obtained using two targeting constructs (Figures 1A,B). For the *NEO* targeting construct, the entire *LPG1* gene was amplified by PCR from *L. infantum* BH46 (MCAN/BR/89/BH46) DNA using *Taq* DNA polymerase (New England Biolabs) and oligodeoxynucleotides AD-358 (forward) (5′-gtacaagcttccatATGGCGCCGCCTCGCTG-3′) and AD-359 (reverse) (5′-gctactcgagTTAGCTGGGGTCAACAG-3′). This fragment was digested with *Hind*III and *Xho*I, and then ligated with the *Hind*III-*Xho*I-digested pBluescript II SK^−^ vector, yielding pBS-*LPG1*. The *NEO* resistance cassette from pLeishNeo (unpublished) was extracted with *Not*I and *Eco*RV, blunted and inserted in the *Msc*I site of pBS-*LPG1*, within the *LPG1* gene, yielding pBS-*LPG1::NEO*. For the *HYG* targeting construct, nucleotides 1–437 of the *LPG1* gene were amplified by RT-PCR from *L. infantum* BH46 mRNA using oligodeoxynucleotides AD-53 (forward) (5′-cgggatccatATGGCGCCGCCTCGCTG-3′) and AD-357 (reverse) (5′-ggaattcTCGGGGTGGTGAATG-3′). This fragment was digested with *Bam*HI and *Eco*RI, and then ligated with the *Bam*HI-*Eco*RI-digested pBluescript II SK^−^ vector. A 467-bp fragment containing nucleotides 781–1,247 of the *LPG1* ORF was amplified by PCR from *L. infantum* BH46 genomic DNA using oligodeoxynucleotides AD-355 (forward) (5′-gcaagcttGGCATCTATTACACAGACCACAAGG-3′) and AD-356 (reverse) (5′-caggtcgacTGGCAGCGAATGTTTTCACC-3′). This fragment was digested with *Hind*III and *Sal*I, then ligated with the same vector, downstream of the first *LPG1* sequence, at the *Hind*III and *Sal*I restriction sites. The *HYG* resistance cassette from pX63-HYG was excised with *Sal*I and *Bam*HI, blunted and inserted between the two *LPG1* sequences, at the *Eco*RV restriction site, yielding pBS-*LPG1::HYG*. For genetic complementation of the Δ*lpg1* mutant, the entire *LPG1* ORF was amplified by PCR from *L. infantum* BH46 genomic DNA using Native *Pfu* polymerase (Stratagene, La Jolla, CA, USA) and oligodeoxynucleotides AD-358 (forward) (5′-gtacaagcttccatATGGCGCCGCCTCGCTG-3′) and AD-359 (reverse) (5′-gctactcgagTTAGCTGGGGTCAACAG-3′). This fragment was digested with *Hind*III and *Xho*I, and then ligated with the *Hind*III-*Xho*I-digested pBluescript II SK^−^ vector, yielding pBSII-*LPG1*. The absence of mutations in the amplified *LPG1* ORF was verified by Sanger sequencing (Génome Québec; GenBank accession No. GU233511). The *LPG1* gene was then excised from pBSII-*LPG1* with *Eco*RV and *Xho*I, blunted and ligated with the *Eco*RV-digested pLeishZeo vector (unpublished), yielding pLeishZeo-*LPG1*.

### Transfection and selection of *L. infantum* Δ*lpg1* promastigotes

Log-phase WT *L. infantum* BA262 (MCAN/BR/89/BA262) promastigotes were first electroporated with the purified *LPG1::HYG* targeting construct (excised as a 2.9-kb *Xba*I-*Kpn*I fragment from pBS-*LPG1::HYG*) using 0.2 cm electroporation cuvettes, at 0.45 kV and a high capacitance of 500 μF as previously described (Turco et al., 1994). Following electroporation, promastigotes were incubated for 24 h in drug-free, complete M199 medium and subsequently grown in the presence of 50 μg/mL Hygromycin B (Roche Diagnostics). To generate *LPG1*-null mutants, log-phase *lpg1*^+/HYG^ heterozygous *L. infantum* BA262 promastigotes were electroporated with purified *LPG1::NEO* targeting construct (excised as a 3.8-kb *Hind*III-*Kpn*I fragment from pBS-*LPG1::NEO*) and grown after 24 h in the presence of both 50 μg/mL Hygromycin B and 70 μg/mL G418 (Life Technologies). Absence of LPG in the resulting double drug-resistant Δ*lpg1* promastigotes was verified by Western blot analysis and confocal immunofluorescence. To restore *LPG1* expression, log-phase *L. infantum* BA262 Δ*lpg1* cells were electroporated with pLeishZeo-*LPG1*. Complemented mutants *(*Δ*lpg1* + *LPG1*) were selected with 80 μg/mL Zeocin (in addition to G418 and Hygromycin B at concentrations specified above) and verified by Western blotting and confocal immunofluorescence.

### Parasite cultures

*L. infantum* promastigotes were cultured in HOMEM medium supplemented with 10% inactivated Fetal Bovine Serum (FBS), 100 U/mL penicillin, 100 μg/mL streptomycin and 2 mM L-glutamine in 25 cm^2^ flasks at 24°C until late log-phase. For *L. infantum* BA262 Δ*lpg1*, Hygromycin (50 μg/mL) and G418 (70 μg/mL) were added to the medium. For *LPG1*-complemented parasites (Δ*lpg1* + *LPG1*), Hygromycin (50 μg/mL), G418 (70 μg/mL), and Zeocin (100 μg/mL) were added to the medium.

### Western blotting

Late log-phase promastigotes were washed with ice-cold PBS containing 1 mM Na_3_VO_4_, then lysed in 50 mM Tris-HCl pH 8, 150 mM NaCl and 1% Nonidet P-40, containing complete protease inhibitors (Roche Applied Science) and phosphatase inhibitors (1 mM Na_3_VO_4_, 50 mM NaF, 1.5 mM EGTA and 10 mM Na_4_P_2_O_7_). Samples were sonicated briefly, and insoluble material was removed by centrifugation for 10 min at 4°C. Protein concentrations were determined using the Pierce BCA protein assay kit (Pierce). Proteins were separated by SDS-PAGE and then transferred to Hybond-LFP PVDF membranes (GE Healthcare Life Sciences) using a Trans-Blot SD Semi-Dry Transfer Cell apparatus (BioRad). Membranes were blocked with 5% BSA and incubated with the mouse monoclonal antibody CA7AE (MediMabs). For immunodetection, goat anti-mouse IgM Heavy Chain Secondary antibody conjugated with horseradish peroxidase (HRP), and enhanced chemiluminescence (ECL) detection reagents from GE Healthcare Life Sciences were used.

### Confocal immunofluorescence microscopy

Late log-phase promastigotes were adhered on Poly-L-Lysine-coated glass coverslips (BD Biosciences, San Jose, CA) by centrifugation, fixed with 4% paraformaldehyde (Canemco and Marivac) for 20 min and simultaneously blocked and permeabilized with a solution of 0.1% Triton X-100, 1% BSA, 6% non-fat dry milk, 20% goat serum and 50% FBS for 20 min. The distribution of LPG and other PGs containing the Gal(β1,4)Man(α1-PO_4_) repeating unit epitope was visualized using the mouse monoclonal antibody CA7AE (MediMabs, 1:2,000) after 2 h incubation followed by Alexa Fluor 568 goat anti-mouse IgM (Molecular Probes) at 1:500 for 30 min incubation. Parasite nuclei were stained with DAPI (Molecular Probes) at 1:17,000. All steps were performed at room temperature. Coverslips were then mounted in Fluoromount-G (Interscience) and sealed with nail polish. Promastigotes were observed with a Plan APOCHROMAT 63x oil-immersion DIC 1.4 NA objective on a Zeiss LSM780 confocal microscope equipped with a 30 mW 405 nm diode laser, 25 mW 458/488/514 argon multiline laser, 20 mW DPSS 561 nm laser and 5 mW HeNe 633 nm laser, coupled to a Zeiss Axio Observer Z1. Images were acquired in plane scanning mode, and were minimally and equally processed using Carl Zeiss ZEN 2011 software.

### Electron microscopy

Late log-phase promastigotes were fixed with 2% paraformaldehyde plus 2.5% glutaraldehyde in 0.1 M phosphate buffer (pH 7.4) overnight at 4°C. Next, parasites were processed for Transmission Electron Microscopy (TEM) by post-fixing in 1% osmium tetroxide (OsO_4_) plus 0.8% potassium ferricyanide in 0.1 M cacodylate buffer, pH 7.2, then dehydrated in acetone at increasing concentrations of 50, 70, 90, and 100% followed by processing for resin embedding (PolyBed 812, Polysciences). Sections were mounted on uncoated 200-mesh copper grids and viewed under a TEM microscope (JEOL JEM-1230). Alternatively, parasites were processed for Scanning Electron Microscopy (SEM) by first fixing as described above, then adhered on Poly-L-Lysine-coated glass coverslips and post-fixed as described above. Samples were then submitted to critical point-drying under CO_2_, coated with a 20 nm-layer of gold particles and examined under SEM (JSM-6390LV, JEOL).

### Parasite growth curves

Early log-phase promastigotes (1 × 10^5^/ml) were cultured and the number of viable promastigotes was determined by daily direct counting performed in a Neubauer chamber.

### Lipid droplets staining and quantification

Late log-phase promastigotes were fixed with 3.7% formaldehyde and stained with osmium tetroxide. Cell morphology was observed, and LD were counted by light microscopy using a 100X objective lens in 50 consecutively scanned parasites (Araújo-Santos et al., 2014).

### Bone marrow-derived macrophages (BMDM) macrophage and infection

Bone marrow-derived macrophages (BMDM) were obtained from C57BL/6 mice as previously described. Briefly, cells were collected from femurs and differentiated in RPMI 1640, 20% inactivated FBS, 30% L929 cell-conditioned media (LCCM), 2 mM L-glutamine, 100 U/mL Penicillin, and 100 μg/mL Streptomycin at 36°C under 5% CO_2_. BMDMs were collected after 7 days and seeded on tissue culture plates in RPMI 1640 media, 10% inactivated FBS, 5% LCCM and 2 mM L-glutamine (Araújo-Santos et al., 2014).

Cells (2 × 10^5^) adhered on coverslips were infected with either WT, Δ*lpg1*, or Δ*lpg1*+*LPG1* parasites at a 10:1 multiplicity of infection (MOI). After 4 or 72 h of infection, coverslips were fixed and stained with DiffQuik (Wright-Giemsa). Intracellular parasites were counted under light microscopy to determine the infection index under each experimental condition (Araújo-Santos et al., 2014).

### RAW 264.7 cell line, culture, and infection

The mouse macrophage leukemia cell line RAW 264.7 (TIB-71; American Type Culture Collection (ATCC), Manassas, VA, USA) was maintained in DMEM medium with high glucose (Vitrocell Embriolife, Campinas, SP, Brazil) supplemented with 10% heat-inactivated fetal bovine serum (Sigma-Aldrich, St. Louis, MO, USA), 100 U/ml penicillin and 100 μg/ml streptomycin in an incubator at 37°C under 5% CO_2_. RAW 264.7 cells were infected with either WT, Δ*lpg1* or Δ*lpg1*+*LPG1* parasites at a 10:1 multiplicity of infection (MOI). After 4 or 8 h of infection, cells were processed for quantitative RT-PCR. For the luciferase reporter assay, cultures were washed 2 h post-infection and analyzed 24 h later.

### RNA extraction and RT-qPCR

For real time quantitative polymerase chain reaction analysis, total RNA of control and infected RAW 264.7 cells (1 × 10^6^ cells) was extracted using an Invitrap® Spin Cell RNA mini kit (STRACTEC Molecular GmbH, Berlin, Germany). RNA extracts (2 μg) were reverse transcribed into first-strand cDNA with ImProm-II (Promega) and oligo(dT) primers in accordance with manufacturer instructions. The following primer DNA sequences were used to determine iNOS mRNA levels: Forward 5′-CAGCTGGGCTGTACAAACCTT-3′ and Reverse: 5′-CATTGGAAGTGAAGCGTTTCG- 3′, while GAPDH mRNA levels were quantified using: Forward 5′-TGCACCACCAACTGCTTAGC-3′ and Reverse 5′-GGCATGGACTGTGGTCATGAG-3′. Amplicon specificity was carefully verified by the presence of a single melting temperature peak in dissociation curves calculated following RT-qPCR, which was performed via the Applied Biosystems StepOne^TM^ detection system (Applied Biosystems) using GoTaq® qPCR Master Mix (Promega Corp., Madison, WI, USA). All RT-qPCR analyses were performed in triplicate. RT-qPCR data was normalized using GAPDH primers as an endogenous control. All gene expression ratios were calculated by the ΔΔCt method using StepOne software version 2.0 (Applied Biosystems).

### Transient transfections and luciferase assays

To investigate NF-κB transcriptional activity, RAW 264.7 were plated in 48-well polystyrene plates (1 × 10^5^ cells per well) and transfected with 1 μg of the p6kB-Luc luciferase reporter construct (kindly provided by Dr. Patrick Baeuerle, Munich University) in the presence of LIPOFECTAMINE 2000 reagent (Invitrogen, Carlsbad, CA, USA). pTK-3XNS luciferase reporter construct was used to measure iNOS promoter activity, provided by Dr. David Geller (University of Pittsburgh, Pennsylvania, EUA). Luciferase activity was normalized using 40 ng of pRL-CMV plasmid (Promega Corp., Madison, WI, USA). Transfected cells were infected with either WT, Δ*lpg1* or Δ*lpg1*+*LPG1* parasites at a 10:1 MOI. After 24 h of infection, cells were washed with PBS, lysed according to the Dual Luciferase System protocol (Promega Corp.), and analyzed in a GloMax®-Multi detection system (Promega Corp.). Positive controls consisting of cells stimulated with 1 μg/mL of LPS (Sigma-Aldrich) were used to induce the activation of iNOS gene expression.

### Statistical analysis

BMDM and RAW 264.7 cell infection assays were performed in triplicate, and each experiment was repeated at least three times. Data are presented as the mean and SE (standard error) of representative experiments, and GraphPad Prism 5.0 software (GraphPad Software) was used for data analysis. Means from different groups were compared by One-way ANOVA and comparisons between two groups were performed using the Student Newman-Keuls post-test. Differences were considered statistically significant when *p* ≤ 0.05.

## Results

### Generation of a *L. infantum Lpg1*-null Δ*lpg1* mutant

To generate a *L. infantum* LPG-defective (Δ*lpg1*) mutant, WT *L. infantum* BA262 promastigotes were transfected with the *LPG1* targeting constructs (Figures 1A,B). The resulting *HYG*- and *NEO*-resistant Δ*lpg1* parasites were transfected with a *LPG1* expression vector to generate add-back LPG-expressing parasites (Δ*lpg1* + *LPG1*). Loss of LPG expression in the Δ*lpg1*, was determined by comparing LPG levels in WT, Δ*lpg1*, and Δ*lpg1* + *LPG1 L. infantum* promastigotes by Western blot and by confocal immunofluorescence microscopy (Figures 1C,D). Together, these data indicate that the *LPG1* gene was successfully deleted in the Δ*lpg1* mutants, resulting in the generation of a LPG-defective *L. infantum* mutant.

### *LPG1*-*null* mutants retain *L. infantum* viability and morphology

**Figure 2:**
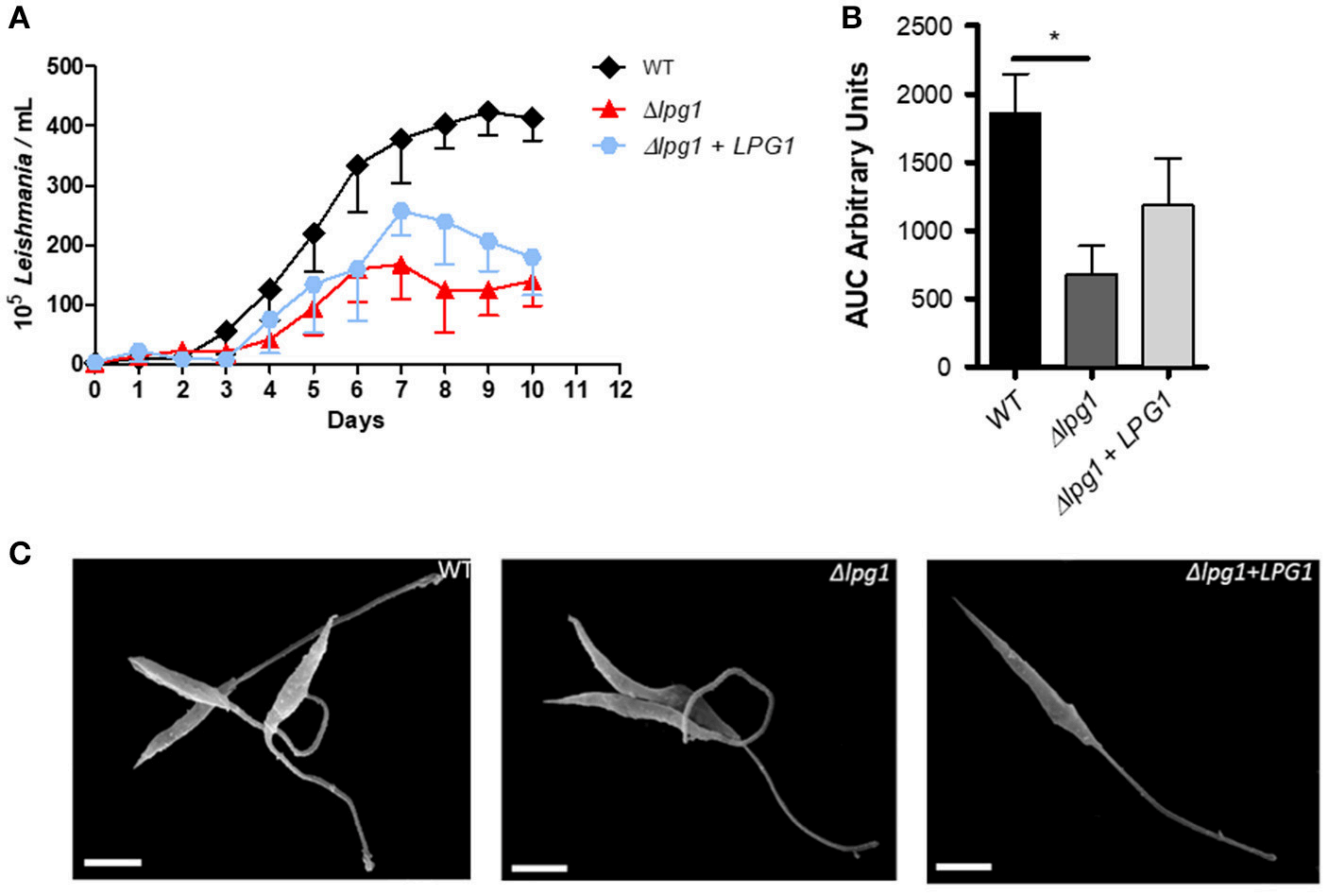
Growth curve and morphology of the Δ*lpg1* mutant. WT, Δ*lpg1* and Δ*lpg1* + *LPG1* parasites were cultured at initial concentrations of 1 × 10^5^/ml in HOMEM medium. **(A)** Axenic growth curve of late log-phase WT, Δ*lpg1* and Δ*lpg1* + *LPG1* parasites, as showed by the area under the curve (AUC) **(B)**. The number of viable parasites was evaluated by direct counting. Each point represents mean and SE. Data are representative of at least three independent assays and were collected in triplicate for each condition. ^*^p < 0.05. **(C)** Parasites were processed for scanning electron microscopy (SEM) and photographed under a JEOL JSM-6390LV microscope at 6000x magnification **(C)**. Scale bar, 2 μm.

**Figure 3:**
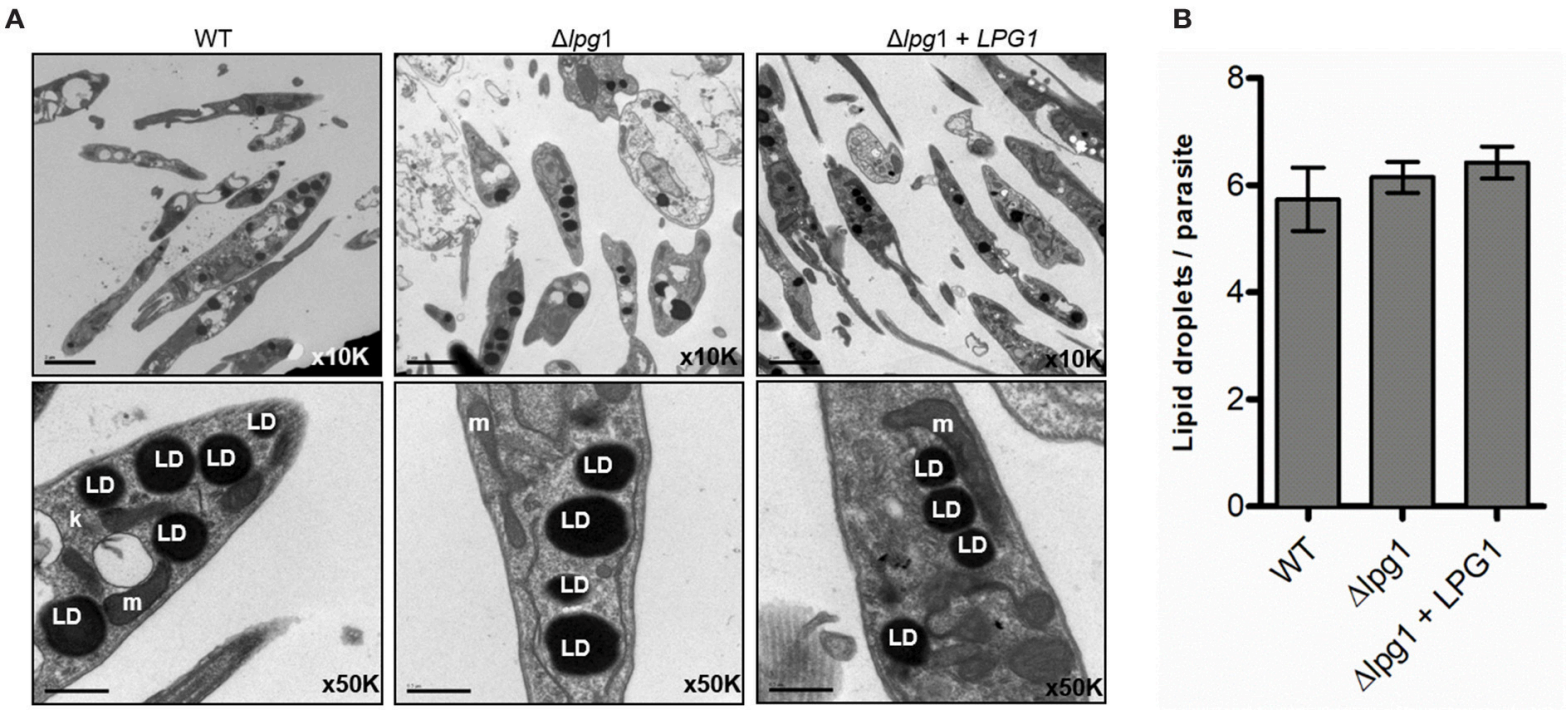
Deletion of *LPG1* does not alter LD formation in *Leishmania infantum*. **(A)** Panels show stationary phase WT, Δ*lpg1* and Δ*lpg1* + *LPG1* promastigotes analyzed by transmission electron microscopy (TEM) and photographed under a JEOL 1230 microscope. **(B)** Bars represent the mean number of LD ± SE in WT, Δ*lpg1* or Δ*lpg1* + *LPG1* parasites stained with osmium tetroxide. k, kinetoplast; LD, lipid droplets; m, mitochondrion. Scale bar, 0.5 μm.

To determine the effect of deleting *LPG1* on parasite growth and morphology, axenic cultures of the three isolates were monitored and counted daily for 10 days until reaching late log phase. A delayed replication capability of the Δ*lpg1* mutant parasites was noted in comparison to the WT and Δ*lpg1* + *LPG1* parasites (Figure 2A). Wild-type *L*. *infantum* presented regular growth for 7 days until reaching stationary phase, with a cell density of approximately 3–4 × 10^7^ parasites/ml, while the Δ*lpg1* mutant reached the same phase approximately 3 days later, with a cell density of 1–2 × 10^7^ parasites/ml (Figure 2A). The Δ*lpg1* + *LPG1* mutants presented an intermediate growth profile, reaching stationary phase shortly after the WT parasites. Area Under the Curve (AUC) analysis of the growth curve revealed a significant difference only when comparing WT and Δ*lpg1* parasites (*p* < 0.05), yet no differences were observed between WT and Δ*lpg1* + *LPG1* mutants (Figure 2B). Upon reaching stationary phase, parasites were examined by electron microscopy to assess the presence of morphological alterations. Under both SEM and TEM, no alterations in morphology (Figure 2C) or in ultrastructural characteristics (Figure 3A) were detected among WT, Δ*lpg1*, and Δ*lpg1* + *LPG1* promastigotes. In addition, the absence of *LPG1* had no impact on the number of lipid bodies present within the parasites (Figure 3A). Hence, whereas the *LPG1* gene had a limited impact on *L. infantum* promastigotes proliferation, it did not significantly alter morphological features of these parasites (Figure 3).

### *Lpg1*-*null* mutants exhibit limited survival in macrophages

**Figure 4:**
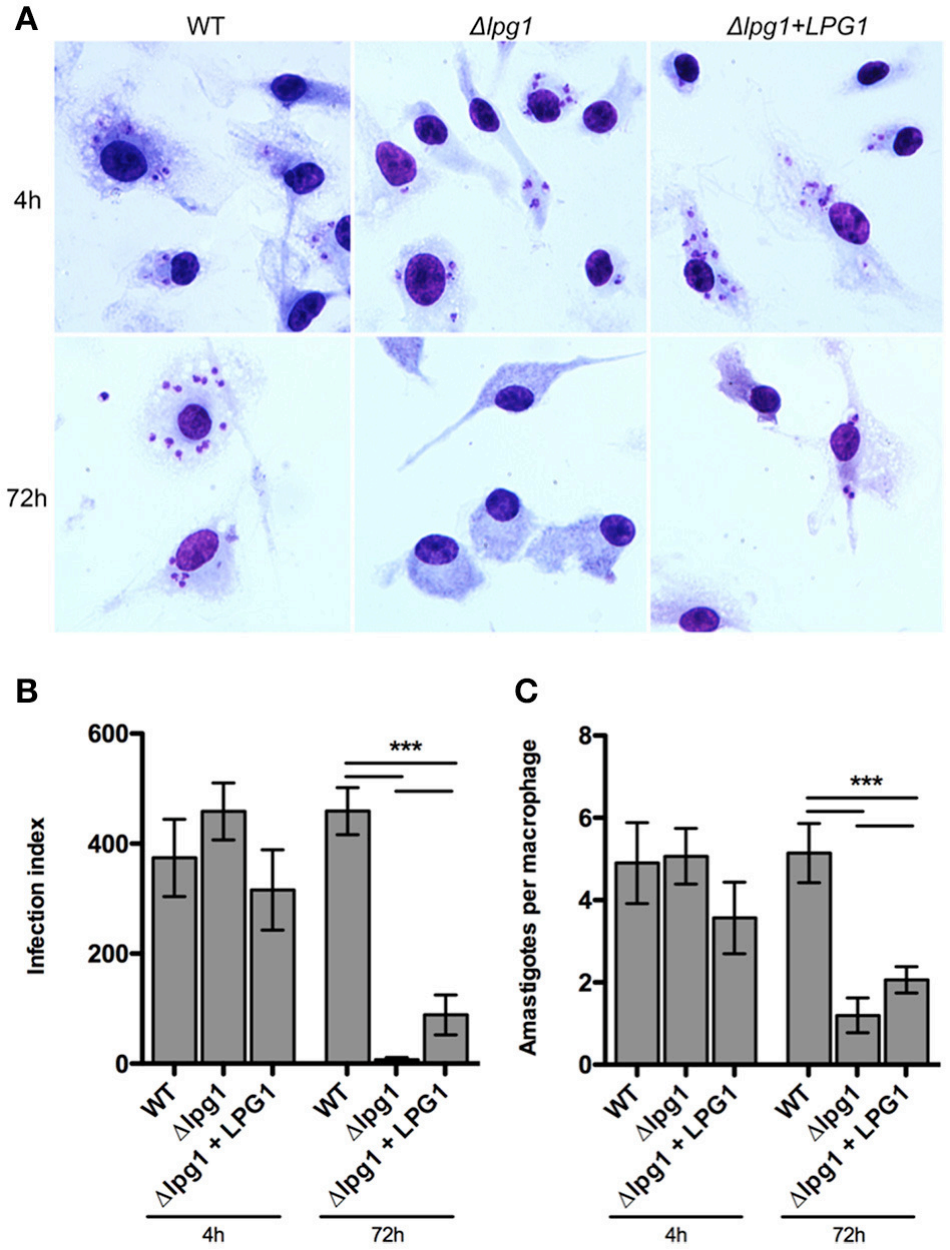
*LPG1* promotes intraphagosomal survival in infected macrophages. C57BL/6 BMDMs were infected with WT, Δ*lpg1* and Δ*lpg1* + *LPG1* promastigotes as described under Materials and Methods. At 4 and 72 h post-infection, cells were fixed and Giemsa-stained. **(A)** Micrographs of infected BMDMs at 4 and 72 h post-infection. The infection index **(B)** and amastigotes per macrophage **(C)** were quantified by light microscopy. Arrows point to amastigotes inside a parasitophorous vacuole. Original magnification × 1000. Statistical differences were evaluated using the Student Newman-Keuls test. ^***^p < 0.001 compared to WT or between groups.

To evaluate differences in parasite survival among WT and transgenic parasites *in vitro*, BMDMs were infected for 4 or 72 h. No differences were observed between the *Leishmania* parasites after 4 h of infection (Figure 4). However, at 72 h post-infection, WT parasites survived more efficiently than the Δ*lpg1* mutant. Expression of *LPG1* in the Δ*lpg1* mutant partially restored its capacity to survive and replicate within macrophages. These data reinforce the importance of LPG as a virulence factor in the successful maintenance of *Leishmania infantum* infection.

### *LPG1*-*null* mutants induce NF-κB-dependent iNOS expression in macrophages

**Figure 5:**
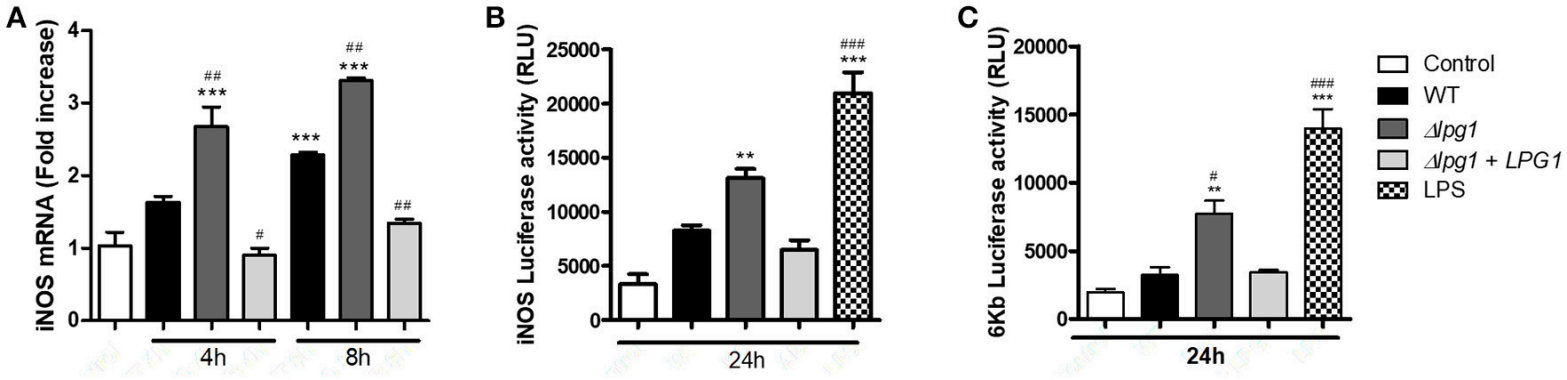
*L. infantum* promastigotes evade NF-κB-dependent iNOS in an LPG-dependent manner in RAW 264.7 cells. RAW 264.7 cells were infected with either WT, Δ*lpg1* or Δ*lpg1* + *LPG1* promastigotes. After 4 or 8 h of infection, iNOS expression was determined by qPCR **(A)**. RAW 264.7 cells were transfected with either the iNOS promoter reporter construct pTK-3XNS, or the NF-κB consensus luciferase reporter construct (p6κB-Luc) prior to infection with either *L. infantum* WT, Δ*lpg1* or Δ*lpg1*+*LPG1*, or stimulation with LPS. At 24 h post-infection, activity of the iNOS promoter **(B)** and the NF-κB reporter **(C)** was measured by quantification of luciferase activity **(B)**. Bars represent means ± SE of three representative experiments performed in triplicate for murine cells. ^***^p < 0.0001; ^**^p < 0.001 compared to control. ^###^*p* < 0.0001, ^##^*p* < 0.001 and ^#^*p* < 0.05 compared to WT. *P* < 0.0001 compared with control group Student Newman-Keuls post-test.

To assess the impact of LPG on the expression of inducible nitric oxide synthase by host cells, we first analyzed iNOS transcript levels in RAW 264.7 cells infected with WT or transgenic parasites. The Δ*lpg1* mutant induced a robust (3.5-fold increase) expression of iNOS compared to WT and Δ*lpg1* + *LPG1* promastigotes (Figure 5A). We next performed luciferase reporter assays to characterize the modulation of the iNOS promoter by *L. infantum* promastigotes. RAW 264.7 cells were transiently transfected with either the iNOS promoter reporter construct pTK-3XNS or the NF-κB consensus luciferase reporter construct (p6κB-Luc) prior to infection with either *L. infantum* WT, Δ*lpg1* or Δ*lpg1*+*LPG1*, or stimulation with LPS. As shown in Figures 5B,C, LPG-deficient promastigotes induced stronger activation of the iNOS promoter and of the NF-κB reporter. Collectively, these findings indicate that LPG contributes to the evasion of iNOS expression by *L. infantum* promastigotes.

## Discussion

Previous studies using purified LPG were important to unravel its impact on the activation of the immune system. Although purified LPG from different species can activate the release of inflammatory mediators, understanding the role of this response in the context of infection remains a challenge. While some groups have characterized Old World *LPG1*-defective *Leishmania* species, the behavior of these parasites when compared to their WT counterparts varies depending on the species under study (Spath et al., 2003; Capul et al., 2007; Forestier et al., 2014). Here, we generated for the first time a LPG-deficient mutant of *L. infantum*, a New World species cluster.

A comparison of parasite growth between axenic cultures containing each of the three isolates showed that the deletion of *LPG1* resulted in a delayed capability of the Δ*lpg1* mutant parasites to replicate in comparison to cultures of WT and Δ*lpg1* + *LPG1* parasites. While the deletion of the *LPG1* gene had a limited impact on *L*. *infantum* promastigote proliferation, no significant morphological or ultrastructural alterations were seen in these parasites, indicating that targeting the *LPG1* gene does not interfere with the intrinsic cell biology of *L. infantum*.

Recently, we have demonstrated that intact LPG from *L. infantum* promastigotes, but not its glycan and lipid moieties, induced a range of pro-inflammatory responses, including prostaglandin E_2_ (PGE_2_) and nitric oxide (NO) release, increased LD formation, and inducible nitric oxide synthase (iNOS) and cyclooxygenase-2 expression (Lima et al., 2017). Consequently, a limitation of using purified LPG is that the physiological conditions present in host cell-parasite interactions are not accurately replicated. LDs are key cytoplasmic organelles involved in production of lipid mediators and pro-inflammatory cytokines in mammalian cells (Bozza et al., 2011). Several intracellular pathogens, including *Leishmania*, take advantage of LD formation in host cells (Rabhi et al., 2016). Moreover, LDs have also been described in trypanosomatids in association with arachidonic acid metabolism (Araújo-Santos et al., 2014). Our group previously reported an increase in LD formation during *L. infantum* metacyclogenesis, as well as in the intracellular amastigote form (Araújo-Santos et al., 2014). Here, we showed that the absence of the *LPG1* gene in *L. infantum* did not alter the biogenesis of LDs. In addition, our previous findings showed that parasite-derived PGF_2α_ produced inside LDs plays a critical role during macrophage infection (Araújo-Santos et al., 2014). We fully intend to comprehensively investigate the potential influence of *LPG1* on the release of PGF_2α_ in infected macrophages using this novel *L. infantum* Δ*lpg1* mutant.

In *L. major* and *L. donovani*, the specific loss of LPG through the ablation of *LPG1* galactofuranosyl transferase strongly impairs the ability of parasites to survive within the sandfly host, as well as to establish infection in mammalian macrophages and in mice (Sacks et al., 2000; Spath et al., 2003; Secundino et al., 2010). Hence, in these species, LPG impairs the microbicidal mechanisms associated with the biogenesis of phagolysosomes, including assembly of the NADPH oxidase and recruitment of the v-ATPase (Lodge et al., 2006; Vinet et al., 2009). Our results indicate that, similarly to these species, *L. infantum LPG1* is required for replication within macrophages. Whether LPG contributes to the ability of *L. infantum* to successfully infect macrophages through the impairment of phagolysosomal biogenesis remains to be investigated. Interestingly, we observed that our Δ*lpg1* mutant parasites induced robust NF-κB-dependent iNOS expression compared to parental WT *L. infantum* promastigotes. This seems to suggest that the reduced survival of Δ*lpg1* mutants in mouse macrophages may be related to higher levels of iNOS, which is responsible for the generation of leishmanicidal nitric oxide (Coelho-Finamore et al., 2011; Passero et al., 2015). Further study will involve investigating the contribution of nitric oxide production with respect to the reduced ability of Δ*lpg1* mutants to survive within macrophages. The underlying mechanism by which Δ*lpg1* mutant parasites induce high levels of NF-κB activation remains unknown, and thus represents an additional aspect of host cell-parasite interplay that we intend to further investigate.

With regard to the partial restoration of the WT phenotype observed in Δ*lpg1* + *LPG1* parasites, it has been well-documented that complemented parasites commonly do not fully recover virulence. The inappropriate regulation of *LPG1* expression by the episomal vector may be a possible explanation for this observation (Späth et al., 2000; Spath et al., 2003; Joshi et al., 2002). A previous study demonstrated that *L. major LPG1*-deficient mutant promastigotes present an attenuated virulence phenotype, as evidenced by the delayed formation of lesions *in vivo* (Späth et al., 2000). In addition, this delay was associated with a 100-fold decrease in parasite survival within macrophages *in vitro*. The data presented herein are consistent with these results, as well as with other reports in the literature (Privé and Descoteaux, 2000; Sacks et al., 2000; Zhang et al., 2004) propounding LPG as a virulence factor.

Taken together, the present findings support the importance of creating LPG-deficient mutants in various *Leishmania* spp. as a unique tool to investigate the specific impact and contribution of this abundant virulence factor in the complex host cell-*Leishmania* interplay. Hence, we are currently conducting studies to compare the responses of various immune cells to live *L. infantum* promastigotes in the presence or absence of surface-expressed LPG, since we feel it is important to thoroughly characterize these isolates to obtain a more comprehensive understanding regarding the role of *L*. *infantum LPG1* in future *in vitro* and *in vivo* studies.

## Author contributions

ML-S, CM, JL, GA, GQ-C, AC, SM-P, UG, LF, TA-S, AD, and VB conceived and designed the study, contributed to the data analysis, drafted, and revised the manuscript. ML-S, CM, JL, GA, GQ-C, AC, SM-P, CF, FJ-S, and TA-S performed the experiments. ML-S, CM, JL, LF, TA-S, AD, and VB wrote and revised the manuscript. All authors read and approved the final version of this manuscript.

### Conflict of interest statement

The authors declare that the research was conducted in the absence of any commercial or financial relationships that could be construed as a potential conflict of interest. The reviewer LM declared a shared affiliation, with no collaboration, with two of the authors, AC and UG, to the handling Editor.
